# Knowledge, Attitudes, and Practices Regarding Infertility Among Lebanese Women Experiencing Difficulty Conceiving

**DOI:** 10.1155/ogi/1745668

**Published:** 2025-12-30

**Authors:** Reva Mosleh, Jana Kassir, Hiba Assi, Hassan Ajami, Joseph Azoury, Roula Ajrouche, Amal Al-Hajje

**Affiliations:** ^1^ École Doctorale des Sciences et Technologies (EDST), Lebanese University, Hadat, Lebanon, ul.edu.lb; ^2^ Clinical and Epidemiological Research Laboratory, Faculty of Pharmacy, Lebanese University, Hadat, Lebanon, ul.edu.lb; ^3^ Hope Clinic, Al Zahraa Hospital University Medical Center, Beirut, Lebanon; ^4^ Azoury In Vitro Fertilization Clinic, Mount Lebanon Hospital, Beirut, Lebanon; ^5^ INSPECT-LB (Institut National de Santé Publique, d’Epidémiologie Clinique et de Toxicologie-Liban), Beirut, Lebanon

## Abstract

**Background:**

Infertility is a significant global challenge, impacting millions of individuals across the world. In Lebanon, where fertility rates are declining, understanding the knowledge, attitudes, and practices of women experiencing infertility is vital to dispelling misconceptions, improving healthcare, and offering better support for those struggling to conceive.

**Methods:**

A cross‐sectional study was conducted between June and September 2024, involving 346 Lebanese women from two fertility centers in Beirut. Participants were randomly selected to complete the questionnaire through individual interviews. Descriptive and bivariate analyses were performed, and generalized linear models were used to explore the influencing factors for each of the knowledge, attitudes, and practice scores.

**Results:**

The results showed that 63.6% of participants had high knowledge, 66.2% had positive attitudes, and 82.1% exhibited good practices. The generalized linear models revealed that being a healthcare provider (*β* = 0.663), sleeping ≥ 7 h (*β* = 0.409), and having a history of anxiety (*β* = 1.258) were associated with higher knowledge scores, while advanced female age as a cause of infertility was linked to lower knowledge (*β* = −0.997). For attitudes, medical insurance (*β* = −1.312) and a family income of 1000–2000 USD (*β* = −2.85) improved attitudes, while smoking (*β* = 3.874), history of endometrial ablation (*β* = 5.506), and longer marriage duration (*β* = 0.135) worsened them. For practices, longer marriage duration (*β* = 0.012) and a previous assisted reproductive technology (ART) experience (*β* = 0.154) improved practices, while having a history of respiratory disorder (*β* = −0.472) decreased practice scores.

**Conclusion:**

This study shows that Lebanese women experiencing difficulty conceiving generally have a high level of knowledge, positive attitudes, alongside good related practices toward infertility. However, knowledge gaps and cultural factors remain, highlighting the need for enhanced reproductive health education and specialized trainings for healthcare providers.

## 1. Introduction

Infertility affects millions of people of reproductive age worldwide and has a significant impact on their families and communities [1]. The World Health Organization (WHO) and the American College of Obstetricians and Gynecologists (ACOG) define infertility as a disease of the male or female reproductive system, characterized by the failure to achieve a pregnancy after 12 months or more of regular unprotected sexual intercourse or therapeutic donor insemination in women under 35 years of age, or within 6 months in women over 35 years of age [1, 2]. The World Health Organization reports that between 48 million couples and 186 million individuals live with infertility globally [1]. According to its report in 2023, approximately 1 in 6 people globally have experienced infertility at some stage in their lives, and the lifetime infertility prevalence is 17.8% for high‐income countries and 16.5% for low‐ and middle‐income countries [3]. Infertility affects both genders equally, with male and female factors each contributing to around 35% of cases, while combined factors account for 20%. The cause remains unexplained in 10% of cases, where couples are often classified as having unexplained infertility [4]. Female infertility is commonly caused by ovulatory disorders, endometriosis, pelvic and tubal adhesions, and uterine or cervical issues, while male infertility can result from endocrine disorders, sperm transport issues, testicular defects, or unexplained factors [5–7]. In Lebanon, a cross‐sectional study reported an infertility rate of 34.3%, but its small sample size and methodological limitations undermine the reliability of the findings, highlighting a gap in comprehensive research on infertility prevalence [8]. Furthermore, according to the United Nations World Population Prospects, fertility rates in Lebanon have significantly decreased, from 5.7 in 1960 to 2.1 in 2018, with a continued decline [9]. Based on global‐metrics datasets, in 2025, Lebanon’s fertility rate stands at 1.99 births per woman, marking a 0.6 decline from 2024. Projections suggest that this downwards trend will continue until 2050 [10].

With a thorough understanding of infertility, it is crucial to explore how people perceive and manage these issues. A scoping review of 12 studies revealed that men and women seeking care in healthcare facilities reported several misunderstandings and information gaps regarding infertility, but most exhibited positive attitudes and practices towards it [11]. In Lebanon, a cross‐sectional study explored public perceptions of infertility, revealing gender disparities, with men more likely to see infertility as grounds for divorce. Misconceptions about its causes were prevalent, particularly the belief that it is mainly a female issue. Higher education correlated with greater openness to alternative family‐building options, though the study’s sample of mainly university students limits the generalizability of its findings [12]. Nevertheless, previous research in Lebanon shows that women’s fertility quality of life is lower compared to Western countries, influenced by factors such as difficulties in conceiving, not having children, and facing societal stigma [13].

Research on infertility often overlooks Lebanon’s unique cultural, social, and economic contexts, highlighting a gap in understanding the general population’s awareness. Addressing this gap is crucial for designing effective educational programs, eliminating misconceptions, raising public awareness, encouraging early intervention, and improving fertility care. This study, therefore, aims to assess the knowledge, attitudes, and practices of Lebanese women experiencing difficulty conceiving regarding infertility and explore the influencing factors affecting each.

## 2. Materials and Methods

### 2.1. Study Design and Sampling Method

This cross‐sectional study was conducted from June 1, 2024, to September 30, 2024, at Hope Clinic (Al Zahraa Hospital University Medical Center) and Azoury IVF (In‐vitro Fertilization) Clinic (Mount Lebanon Hospital) in Beirut. A simple random sampling method was employed to recruit participants from the clinics’ waiting rooms. Eligible individuals were approached and invited to participate, and those who consented were individually interviewed by one of three trained researchers—one PhD candidate and two Master’s students in Clinical Research and Pharmacoepidemiology—using a structured paper questionnaire. Participation was anonymous and voluntary, with a written consent obtained. Interviews were conducted in Arabic and took 12 to 15 min to complete.

### 2.2. Inclusion and Exclusion Criteria

The study included Lebanese married women of reproductive age (≥ 18 years), actively attempting to conceive, and diagnosed with infertility regardless of the infertility source. Infertility was defined as the inability to conceive after one year of unprotected intercourse for women under 35 years, or after six months for women 35 and older. Exclusion criteria were women with incomplete questionnaire.

### 2.3. Sample Size

The sample size was calculated using Epi Info software [14]. A 95% confidence interval, 5% margin of error, and an expected infertility prevalence of 34.3% resulted in a target sample size of 346 participants [8].

### 2.4. Study Tool

The questionnaire was developed following a comprehensive review of the literature and was structured into three main sections. The first section included 23 questions addressing sociodemographic (residence, medical insurance, income, educational level etc.) and lifestyle factors (smoking, alcohol consumption, exercise etc.) [15]. The second section included 16 questions on health status, covering chronic conditions (hypertension, diabetes, dyslipidemia etc.), gynecological history (menstruation, history of pregnancy, history of gynecological surgery etc.), and infertility history (e.g., causes and family history) [6, 7, 16, 17]. The third section contained 11 questions assessing knowledge of infertility (with responses of “Yes,” “No,” or “I Don’t Know”), 8 questions evaluating attitudes towards infertility (using a Likert scale), and 6 questions examining practices related to infertility (with responses of “Yes,” “No,” or “I Don’t Know”) [12, 18–21]. The questionnaire was translated from English to Arabic and back‐translated to ensure accuracy. Content validity was assessed by experienced professors, and a pilot study with 10–15 participants led to minor revisions.

### 2.5. Data Analysis

The data from the questionnaire were analyzed using SPSS Version 26, with descriptive statistics, bivariate, and multivariate analysis. Frequencies were calculated for categorical variables, and means and standard deviations were used for continuous variables. The normality of continuous variables was assessed both graphically and statistically through skewness and kurtosis [22]. Bivariate analysis of qualitative variables was conducted using the independent samples *t*‐test or one‐way ANOVA with normally distributed score, and the Kruskal–Wallis or Mann–Whitney *U* test with non‐normally distributed score. For continuous variables, Pearson correlation was used for normal data, and Spearman correlation for non‐normal data.

A generalized linear model (GLM) was employed to analyze the data, as it allows for the inclusion of outcome variables that are not normally distributed and can accommodate categorical predictors with multiple categories. GLM was used to identify the significant influencing factors for each of the knowledge, attitudes, and practice score, including variables with *p* values < 0.2. A linear scale was applied for normally distributed data, while a gamma log‐linear scale was used for non‐normally distributed data. *p* values < 0.05 were considered statistically significant. Cronbach’s alpha was used to assess the internal consistency of the scores. While Cronbach’s alpha values of 0.7 and above are recommended [23], a value of 0.6 or higher can still be acceptable especially in exploratory studies and social sciences research [24]. In this study, items with a Cronbach’s alpha of 0.6 or above were considered to have acceptable reliability. The alpha values for the knowledge, attitudes, and practices scores were 0.67, 0.66, and 0.60, respectively.

### 2.6. Scoring

Individual scores for knowledge, attitudes, and practices related to infertility were calculated and used as dependent variables in analyses, with cut‐off points set using Bloom’s taxonomy [25]. For the 11‐question knowledge score, correct answers were assigned a value of 1, while incorrect or “I don’t know” responses were coded as 0. The score ranged from 0 to 11, with 0–6 indicating low knowledge, 7‐8 indicating moderate knowledge, and 9–11 indicating high knowledge. The attitudes score was based on an 8‐question, five‐point Likert scale, ranging from 1 (“*strongly disagree*”) to 5 (“*strongly agree*”). The total score ranged from 8 to 40, with 8–23 indicating a positive attitude, 24–31 a neutral attitude, and 32–40 a negative attitude. For the 6‐question practices score, “yes” response was coded as 1, and “no” or “don’t know” responses were coded as 0. The total score ranged from 0 to 6, with 0–2 indicating poor practice, 3‐4 indicating moderate practice, and 5‐6 indicating good practice.

### 2.7. Ethical Consideration

This study employed a questionnaire for data collection, without invasive procedures or interventions. The study protocol, survey, and consent forms were reviewed and approved by the Institutional Review Board of the School of Pharmacy at the Lebanese University (7/24/D). Data were anonymous, nonidentifiable, and stored according to the University’s data protection guidelines. Written informed consent was obtained, and participants were informed that their involvement was voluntary and they could withdraw at any time without justification.

## 3. Results

### 3.1. Sociodemographic Characteristics and Health Status of the Participants

The study included 346 women, with a mean age of 34 years (±6.48 years). Of these, 53.47% were from Azoury clinic and 46.5% were from Hope clinic. Half of the participants are employed, 53.47% lived in center areas (Beirut and its surrounding districts), and 57.51% had health insurance. Additionally, 90.46% were noncigarette smokers, 63.29% were non‐nargileh smokers (i.e., participants who do not smoke water pipe, but may smoke other tobacco products, or not smoke at all), and 88.44% were nonalcohol consumers. For the past medical history, 38.2% had at least one chronic disease, with obesity (53.78%) and thyroid disorders (25%) being the most common. Among them, 34.39% had routine Pap smears, and 52.02% had at least one previous gynecological surgery, with cesarean section (38.89%) and laparoscopy (36.67%) being the most frequent. Difficulty conceiving was primarily due to female factors (41.91%), such as polycystic ovary syndrome (PCOS) (36.56%), ovulatory disorders (33.10%), and endometriosis (28.97%). Male factors mainly included low sperm count (93.75%) and low sperm quality (92.50%). Also, 9.83% had a family history of infertility, predominantly on the mother’s side (52.94%) (Table 1).

**Table 1:** Sociodemographic characteristics and health status of the participants (*N* = 346).

Variable	Frequency (%)	Mean ± SD
Age (years)		33.82 ± 6.48
BMI (kg/m^2^)		26.19 ± 4.77
Region		
Mount Lebanon	113 (32.66%)	
Beirut	85 (24.57%)	
Beqaa	64 (18.50%)	
South	46 (13.29%)	
North	30 (8.67%)	
Nabatiyeh	8 (2.31%)	
Family monthly income		
< 250 USD	68 (19.65%)	
> 250–500 USD	119 (34.39%)	
> 500–1000 USD	64 (18.50%)	
> 1000–2000 USD	35 (10.12%)	
> 2000 USD	60 (17.34%)	
Educational level		
Didn’t receive any education	4 (1.16%)	
Elementary or intermediate	42 (12.14%)	
High school	61 (17.63%)	
University degree	180 (52.02%)	
Postgraduate degree	59 (17.05%)	
Past medical history of chronic disease	132 (38.20%)	
Infertility cause		
Female factors	145 (41.9%)	
Male factors	80 (23.10%)	
Combined factors	80 (23.10%)	
Unexplained factors	71 (20.50%)	

*Note: N* = frequency.

Abbreviations: BMI, body mass index; SD, standard deviation; USD, United States dollar.

### 3.2. Description of the Scores

#### 3.2.1. Knowledge Score

The results showed that the mean knowledge score was 8.73 ± 1.77 (median = 9; range = 0–11). Of the participants, 220 (63.6%) had high knowledge, 82 (23.7%) had moderate knowledge, and 44 (12.7%) had poor knowledge (Table 2).

**Table 2:** Knowledge items.

Knowledge questions towards infertility	Yes *N* (%)	No *N* (%)	I don’t know *N* (%)
Is infertility a disease?	135 (39.0%)	154 (44.5%)	57 (16.5%)
Can infertility be treated medically?	297 (85.8%)	19 (5.5%)	30 (8.7%)
Infertility is defined as the inability to achieve pregnancy after 1 year of regular sexual intercourse?	210 (60.7%)	97 (28.0%)	39 (11.3%)
Should both women and men be examined in cases of infertility?	338 (97.7%)	4 (1.2%)	4 (1.2%)
For a woman who is having difficulty getting pregnant, should visiting a gynecologist be the first option?	338 (97.7%)	4 (1.2%)	4 (1.2%)
If a couple was able to have children before, will they have a chance to face problems conceiving again?	305 (88.2%)	21 (6.1%)	20 (5.8%)
Is the midcycle the most fertile period in a woman’s menstrual cycle?	224 (64.7%)	60 (17.3%)	62 (17.9%)
Does fertility decrease as the female gets older?	323 (93.4%)	8 (2.3%)	15 (4.3%)
Do you know that hormonal imbalances and problems in the female reproductive system can cause female infertility?	287 (82.9%)	20 (5.8%)	39 (11.3%)
Do you know that hormonal imbalances and problems in the male reproductive system can cause male infertility?	278 (80.3%)	22 (6.4%)	46 (13.3%)
Do you know that some causes of infertility remain unknown?	286 (2.7%)	32 (9.2%)	28 (8.1%)

#### 3.2.2. Attitudes Score

The results showed that the mean attitudes score was 19.2 ± 5.16 (median = 19; range = 8–40). Of the participants, 284 (82.1%) had positive attitudes, 54 (15.6%) had neutral attitudes, and 8 (2.3%) had negative attitudes (Table 3).

**Table 3:** Attitudes items.

Attitudes questions towards infertility	Strongly disagree *N* (%)	Disagree *N* (%)	Neutral *N* (%)	Agree *N* (%)	Strongly agree *N* (%)
If a female cannot have a baby, do you think this is a valid reason for divorce?	138 (39.9%)	118 (34.1%)	48 (13.9%)	30 (8.7%)	12 (3.4%)
If a female cannot have a baby, do you think this is a valid reason for the man to have a second marriage?	121 (35.0%)	106 (30.6%)	58 (16.8%)	45 (13%)	16 (4.6%)
If a male cannot have children, do you think this is a valid reason for divorce?	144 (41.6%)	126 (36.4%)	42 (12.1%)	21 (6.1%)	13 (3.8%)
In case of infertility, do you think that the wife should be the only one blamed?	162 (46.8%)	134 (38.7%)	19 (5.5%)	19 (5.5%)	12 (3.5%)
In case of infertility, do you think that the husband should be the only one blamed?	172 (49.7%)	143 (41.3%)	24 (7.0%)	2 (0.6%)	5 (1.4%)
In case of infertility, do you think that both the wife and husband should be blamed?	97 (28%)	80 (23.1%)	47 (13.6%)	79 (23%)	43 (12.4%)
Do you think adoption is unacceptable in our society?	37 (10.7%)	62 (17.9%)	53 (15.3%)	81 (23.4%)	113 (32.7%)
Not having a child decreases the overall quality of life and sense of well‐being?	48 (13.9%)	67 (19.3%)	36 (10.4%)	82 (23.7%)	113 (32.7%)

#### 3.2.3. Practice Score

The results showed that the mean practice score was 4.8 ± 1.31 (median = 5; range = 0–6). Of the participants, 229 (66.2%) had good practices, 97 (28%) had moderate practices, and 20 (5.8%) had poor practices (Table 4).

**Table 4:** Practice items.

Practice questions towards infertility	No *N* (%)	Yes *N* (%)	I don’t know *N* (%)
Did you resort to a treatment to improve fertility?	53 (15.3%)	291 (84.1%)	2 (0.6%)
Did you use complementary and alternative medicine (herbs and vitamins) to improve fertility?	116 (33.5%)	227 (65.6%)	3 (0.9%)
Do enhancing lifestyle habits like quitting smoking or quitting alcohol improve fertility?	54 (15.6%)	261 (75.4%)	31 (9.0%)
Does your husband take medications to improve fertility if necessary?	56 (16.2%)	283 (81.8%)	7 (2.0%)
Do you consider corrective surgery to improve fertility if needed?	25 (7.2%)	306 (88.5%)	15 (4.3%)
Is it okay for your husband to consider corrective surgery to improve fertility if needed?	36 (10.4%)	286 (82.7%)	24 (6.9%)

### 3.3. Bivariate Analysis

#### 3.3.1. Knowledge Score

Higher mean knowledge scores were significantly linked to a history of gynecological surgery (*p* < 0.001), previous laparoscopy (*p* = 0.001), routine Pap smear screening (*p* = 0.002), being a healthcare provider (*p* = 0.016), a family history of infertility (*p* = 0.037), and previous ART use (*p* = 0.016). However, obesity (*p* = 0.048) and advanced female age (*p* = 0.02), reported as infertility causes, were significantly associated with lower mean score. Nonalcohol consumption (*p* = 0.038) and non‐nargileh smoking (*p* = 0.027) were associated with higher scores. Nargileh use frequency (*p* = 0.014) and BMI (*p* = 0.034) had negative correlations with knowledge score (see Supporting Information (available here)).

#### 3.3.2. Attitudes Score

Higher mean attitudes score and hence poorer attitudes towards infertility were significantly associated with different fertility clinics (*p* < 0.001), lack of health insurance (*p* < 0.001), dyslipidemia (*p* = 0.049), female lifestyle habits reported as a female infertility cause (*p* = 0.034), low income (*p* = 0.002), smoking (*p* = 0.043), nonalcoholic status (*p* = 0.03), higher cigarette consumption (*p* = 0.022), and longer marriage duration (*p* = 0.016) (see Supporting Information).

#### 3.3.3. Practice Score

Higher mean practice scores were significantly associated with different fertility clinics (*p* = 0.004), routine Pap smear screening (*p* = 0.006), history of gynecological surgery (*p* = 0.014), history of laparoscopy (*p* = 0.002), history of previous ART uptake (*p* < 0.001), history of previous pregnancy achieved after fertility treatment (*p* = 0.004), different regions (*p* = 0.008), and duration of marriage (*p* = 0.004). However, the frequency of cigarette (*p* = 0.032) and nargileh (*p* = 0.032) smoking were negatively correlated with practice scores (see Supporting Information).

### 3.4. Multivariate Analysis

The results from the first GLM, with the knowledge score as the dependent variable, showed that being a healthcare provider increases the knowledge score by 0.663 points. Similarly, sleeping ≥ 7 h (*β* = 0.409) and having a medical history of anxiety (*β* = 1.258) were significantly associated with a higher knowledge score. However, advanced female age as a cause of female (*β* = −0.997) infertility was significantly associated with lower knowledge score.

The second GLM analysis, with attitudes score as the dependent variable, showed that having medical insurance (*β* = −1.312) and a family higher income (*β* = −2.85) significantly decrease the attitudes score and hence improve the attitudes towards infertility. However, having a history of endometrial ablation (*β* = 5.506), being a cigarette smoker (*β* = 3.874), and increased duration of marriage (*β* = 0.135) significantly worsen the attitudes towards infertility.

A third GLM, with the practice score as the dependent variable, showed that the duration of marriage (*β* = 0.012) and history of previous ART utilization (*β* = 0.154) uptake improve the practices towards infertility. However, having a history of asthma or chronic obstructive pulmonary disease (COPD) (*β* = −0.472) and being a patient at Hope clinic (*β* = −0.12) decline the practice scores towards infertility (Table 5).

**Table 5:** Multivariate analysis.

Influencing factors	Coefficient *β*	SE	95% CI		*p* value
			Lower	Upper	
*Model 1: Generalized linear model taking the knowledge score as the dependent variable*					
Being a healthcare provider	0.663	0.254	0.166	1.161	0.009
Sleeping hours ≥ 7 h	0.409	0.184	0.048	0.769	0.026
Having a medical history of anxiety	1.258	0.587	0.108	2.407	0.032
Advanced female age (as an infertility cause)	−0.997	0.352	−1.687	−0.306	0.005

*Model 2: Generalized linear model taking the attitudes score as the dependent variable*					
Having medical or health insurance	−1.312	0.549	−2.387	−0.237	0.017
Having a history of endometrial ablation	5.506	2.74	0.136	10.876	0.044
Family monthly income (> 1000–2000 USD)	−2.85	1.064	−4.936	−0.765	0.007
Being a cigarette smoker	3.874	1.495	0.943	6.804	0.01
Duration of marriage	0.135	0.061	0.017	0.254	0.025

*Model 3: Generalized linear model with gamma log link taking the practice score as the dependent variable*					
Hope clinic	−0.12	0.056	−0.23	−0.011	0.03
Having a medical history of asthma or COPD	−0.472	0.157	−0.779	−0.165	0.003
Duration of marriage	0.012	0.006	0.001	0.024	0.04
History of previous ART utilization	0.154	0.062	0.032	0.275	0.013

### 3.5. Correlation Between the Knowledge, Attitudes, and Practice Scores

Knowledge of infertility significantly improves the practice score towards infertility, and vice versa (*p* < 0.001), but has no impact on attitudes. Also, the practice score significantly improves attitudes towards infertility (*p* = 0.047), while attitudes do not affect the practice score (Table 6).

**Table 6:** Correlation between the knowledge, attitudes, and practice scores.

Dependent variable	Independent variable	Coefficient *β* ^∗^	*p* value	CI
Infertility knowledge score	Infertility attitudes score	−0.005	0.785	[−0.04, 0.03]
	Infertility practice score	0.470	< 0.001	[0.34, 0.60]

Infertility attitudes score	Infertility knowledge score	−0.043	0.785	[−0.35, 0.26]
	Infertility practice score	−0.418	0.047	[−0.83, −0.01]

Infertility practice score	Infertility knowledge score	0.051	< 0.001	[0.03, 0.07]
	Infertility attitudes score	−0.005	0.137	[−0.01, 0.01]

^∗^GLM with a linear scale for normally distributed scores and a gamma log link for non‐normally distributed ones.

## 4. Discussion

The study revealed that the majority of the participants had high knowledge on infertility. This contrasts with studies in India, Pakistan, and Saudi Arabia, where limited knowledge and misconceptions prevailed [19, 20, 26]. Women showed good awareness of infertility’s treatability, the importance of evaluating both partners, and the role of age, consistent with findings from other studies [20, 26]. This suggests that despite cultural differences, there is widespread recognition of infertility’s medical aspects, likely influenced by improved healthcare access and awareness campaigns. Even so, a significant proportion failed to identify infertility as a disease, a trend also observed in studies from India, Pakistan, and Indonesia [19, 26, 27]. It indicates a strong stigma around infertility, with individuals reluctant to label themselves as “ill.” The historical medicalization of infertility, which only became formally recognized as a disease in 1992, and later officially defined as such later by WHO, may still be influencing how people perceive the condition [1, 28].

Many women had difficulty defining infertility or identifying its causes, reflecting communication gaps likely linked to cultural taboos, limited awareness, and restricted access to reliable information, consistent with other studies [27, 29, 30]. Participants showed some awareness of the most fertile period, similar to a study in India possibly due to personal experience, unlike the general population [26].

When testing for influencing factors of increased knowledge, it was found that healthcare providers scored significantly higher scores compared to nonhealthcare providers, likely due to their exposure to reproductive health information and patient care. However, a study in the United Kingdom reported no such difference [31]. Those who slept less than 7 h had lower knowledge scores, as sleep affects cognitive performance. A study found that 7 h of sleep per day is linked to optimal cognitive performance, with declines observed for durations shorter or longer than this [32]. Those with a history of anxiety tended to have higher knowledge scores, as anxiety may drive individuals to seek more information to alleviate their concerns. This aligns with findings from a Canadian study, which reported that individuals who searched the internet for infertility information were more likely to be women and to experience higher levels of distress [33]. Conversely, identifying advanced age as a cause of infertility was associated with lower knowledge scores, possibly because individuals who perceive age as a major factor may feel less hopeful and therefore less motivated to seek out information or solutions.

The study reveals that attitudes toward infertility in Lebanon are generally positive, a trend that mirrors the findings of a similar study conducted in 2020 [12], while contrasts with more negative perceptions observed in other countries [19, 20, 26]. Even though most participants in Lebanon displayed positive attitudes, which is notably different from regions where infertility is often viewed with stigma, influenced by cultural and societal expectations [34]. Many rejected infertility issues as a reason for divorce, reflecting a shift toward viewing it medically rather than personally. Male infertility carried less stigma in Lebanon, though cultural attitudes vary. Remarriage is more accepted in Saudi Arabia and Pakistan, while polygamy is discouraged in Lebanon [19, 20].

The study revealed that participants generally reject blaming one partner exclusively for infertility, supporting shared responsibility, which contrasts with other findings where women are often blamed [19]. Adoption is controversial in Lebanon due to religious diversity, which influences views on family and child‐rearing.

Financial factors, like health insurance and higher income, were linked to more positive attitudes toward infertility, due to better treatment access hope. In contrast, those without insurance or with lower incomes had more negative attitudes due to limited access and high treatment costs. These findings align with previous study in Lebanon [13]. Smokers had more negative attitudes toward infertility, likely due to smoking’s adverse effects on reproductive health. Besides this, women with longer marriages exhibited more negative attitudes, likely due to prolonged infertility, a finding similar to a study on Indian women that showed a negative association between marital duration and fertility quality of life [35].

The majority of participants demonstrated good practices in infertility management, similar to findings from previous research in Lebanon [18]. Such reflects a high level of awareness and active engagement in appropriate healthcare measures. This trend is consistent with other study, where the use of alternative medicine practices is commonly reported among individuals managing infertility [20].

Lifestyle changes were widely believed to improve reproductive health, aligning with global evidence [36]. Despite this belief, actual engagement in healthy behaviors like physical exercise, smoking cessation, and reducing caffeine intake was lower than expected. This demonstrates the need for more effective counseling to optimize fertility outcomes. The study highlighted that women participants often mentioned infertility as a shared challenge between both men and women in Lebanon. Most of these women were willing to undergo medical treatments, and corrective surgeries.

In the search for influencing factors, previous experience with ART and longer duration of marriage were linked to significantly higher practice scores, reflecting greater engagement and proactivity in infertility management. Practices varied across fertility clinics, highlighting differences in perspectives and approaches. Additionally, those with a medical history of respiratory problems had lower scores, likely due to the physical limitations.

The study found a significant positive relationship between knowledge and practice, indicating that individuals with more knowledge about infertility tend to engage more in infertility‐related practices. However, attitudes toward infertility were not significantly influenced by knowledge, suggesting that while knowledge is shaped by external factors, attitudes are impacted by other variables. A significant relationship was also observed between practice and attitudes, with more active involvement in infertility management leading to more positive attitudes.

This study serves as an assessment of knowledge, attitudes, and practices towards infertility among Lebanese women experiencing difficulty conceiving. The study’s cross‐sectional design is a limitation, as it cannot establish causality and only generate hypotheses. Although women were from various regions, recruiting solely from Beirut clinics may introduce selection bias and limit the generalizability of the findings. Information bias is possible due to the sensitive nature of some questions which could lead to underreporting. Overreporting might also occur, as those who are already willing to undergo ART may have more knowledge, optimism, and higher expectations. Additionally, the KAP scale was not validated with confirmatory factor analysis (CFA), which may affect the accuracy and generalizability of the findings.

## 5. Conclusion

While knowledge scores were high, gaps remained in understanding infertility’s definition and causes. Attitudes were positive, though cultural and religious influences shaped views on adoption and gender roles. Practices showed a strong reliance on medical (infertility medications and corrective surgeries) and complementary treatments (herbs and vitamins).

Strengthening reproductive health education, providing specialized training for healthcare providers, and developing reliable online resources are recommended. Future studies should investigate cultural practices such as polygamy and remarriage related to infertility, compare infertile and fertile individuals, and include CFA to further verify the construct validity of the KAP scale.

## Ethics Statement

The research has received approval from the institutional review board of the School of Pharmacy at the Lebanese University. If accepted, this article will not be published elsewhere in the same form, in English or any other language, without written consent from the copyright holder.

## Disclosure

All authors have approved the final manuscript for submission.

## Conflicts of Interest

The authors declare no conflicts of interest.

## Author Contributions

Conception and study design: Roula Ajrouche, Reva Mosleh, Jana Kassir, and Hiba Assi.

Acquisition of data: Jana Kassir, Hiba Assi, and Reva Mosleh.

Analysis and interpretation of data: Jana Kassir and Reva Mosleh.

Writing original draft: Reva Mosleh and Jana Kassir.

Revising and editing: Amal Al‐Hajje, Joseph Azoury, and Hassan Ajami.

Reva Mosleh and Jana Kassir contributed equally to this work.

## Funding

No funding was received for this manuscript.

## Supporting Information

The supporting information provides the bivariate analysis tables evaluating participants’ knowledge, attitudes, and practices toward infertility. It is referenced in the main text to provide additional detail supporting the reported findings (in‐text citation: see supporting information).

## Supporting information


**Supporting Information** Additional supporting information can be found online in the Supporting Information section.

## Data Availability

The data that support the findings of this study are available on request from the corresponding author. The data are not publicly available due to privacy.
