# Supplementary material for: Knowledge, Attitudes, and Practices Regarding Infertility Among Lebanese Women Experiencing Difficulty Conceiving
Source: Obstet Gynecol Int. 2025 Dec 30;2025:1745668. doi: 10.1155/ogi/1745668 (PMC12767415; doi:10.1155/ogi/1745668)
Supplement: Supplementary file 1 — Supporting Information Additional supporting information can be found online in the Supporting Information section. [file OGI-2025-1745668-s001.docx]

**Supplementary material:**

**Bivariate analysis of factors associated with knowledge score towards infertility**

| **Qualitative variables** | **Mean* ± SD** | **p-value** |
| --- | --- | --- |
| Health care provider (Y) | 9.18 ± 1.42 | 0.016 |
| Pap smear routinely (Y) | 9.14 ± 1.74 | 0.002 |
| Gynecological surgery (Y) | 9.05 ± 1.65 | <0.001 |
| History of laparoscopy (Y) | 9.36 ± 1.55 | 0.001 |
| Family history of infertility (Y) | 9.23 ± 1.39 | 0.037 |
| History of previous ART (Y) | 8.93 ± 1.65 | 0.016 |
| Obesity as a female cause of infertility (N) | 8.83 ± 1.71 | 0.048 |
| Advanced female age as a female cause of infertility (N) | 7.91 ± 1.84 | 0.02 |
| Nargileh Smoker** |  | 0.033 |
| Non-smoker | 8.94 ± 1.75 |  |
| Ex-smoker | 8.76 ± 1.74 |  |
| Smoker | 8.4 ± 1.79 |  |
| Alcohol consumption** |  | 0.01 |
| Non-alcoholic | 8.63 ± 1.8 |  |
| Ex-alcoholic | 9.48 ± 1.43 |  |
| Alcoholic | 9.64 ± 1.77 |  |
| **Qualitative variables** | **Rs** | **p-value** |
| BMI | -0.114 | 0.034 |
| Nargileh sessions per week | -0.131 | 0.014 |

Abbreviations: Y = Yes, N = No, Rs = Spearman correlation coefficient

*Independent samples t-test and One-way ANOVA

**Post-hoc analysis: Nargileh smoker (non-nargileh vs nargileh p=0.027, non-nargileh vs ex-nargileh p=1), Alcohol consumption (non-alcoholic vs ex-alcoholic p=0.038, alcoholic vs non-alcoholic p=0.187)

Variables that lacked significance with the knowledge score (duration of marriage p=0.267, family income p=0.207, region p=0.121, educational level p=0.078, cigarette smoking p=0.684, living area p=0.083, Azoury/hope clinic p=0.092, health insurance p=0.521, occupation p=0.238, physical exercise p=0.61).

**Bivariate analysis of factors associated with attitudes score towards infertility**

| **Qualitative variables** | **Mean* ± SD** | **p-value** |
| --- | --- | --- |
| Clinic name |  | <0.001 |
| Azoury | 18.1 ± 5.26 |  |
| Hope | 20.5 ± 4.77 |  |
| Health insurance (N) | 20.35 ± 5.5 | <0.001 |
| History of dyslipidemia (Y) | 23 ± 5 | 0.049 |
| Life style habits as female cause of infertility (Y) | 22.6 ± 5.4 | 0.034 |
| Family income** |  | 0.002 |
| <250 USD | 20.87 ± 5.18 |  |
| >250 - 500 USD | 19.58 ± 4.5 |  |
| >500 - 1000 USD | 18.97 ± 6.244 |  |
| >1000 - 2000 USD | 17.14 ± 4.76 |  |
| >2000 USD | 18 ± 4.76 |  |
| Cigarette smoking** |  | 0.043 |
| Non-smoker | 18.99 ± 5 |  |
| Ex-smoker | 19.5 ± 3.964 |  |
| Smoker | 21.68 ± 6 |  |
| Alcohol consumption** |  | 0.03 |
| Non-alcoholic | 19.46 ± 5 |  |
| Ex-alcoholic | 17.14 ± 5.8 |  |
| Alcoholic | 17.27 ± 5.2 |  |
| **Qualitative variables** | **Rs** | **p-value** |
| Number of cigarettes smoked/ day | 0.123 | 0.022 |
| Duration of marriage | 0.129 | 0.016 |

* Independent samples t-test and One-way ANOVA

**Post-hoc analysis: Family income (>1000-2000 vs <250 USD p=0.005 and >2000 vs <250 USD p=0.015, >250-500 vs <250 USD p=0.955, <250 vs >500-1000 USD p= 0.321), Cigarette smoking (cigarette smoker vs non-cigarette smoker p=0.037, cigarette smoker vs ex-cigarette smoker p=1), Alcohol consumption (non-alcoholic vs ex-alcoholic p=0.06, non-alcoholic vs alcoholic p=0.494).

Variables that lacked significance with the attitudes score (age p=0.538, BMI p=0.708, region p=0.206, living area p=0.882, educational level p=0.186, nargileh smoking p=0.793, being a healthcare provider p=0.083, occupation p=0.079, physical exercise p=0.303).

**Bivariate analysis of factors associated with practice score towards infertility**

| **Qualitative variables** | **Mean Rank*** | **p-value** |
| --- | --- | --- |
| Clinic name |  |  |
| Azoury | 187.34 | 0.004 |
| Hope | 157.59 |  |
| Pap smear routinely (Y) | 192.97 | 0.006 |
| History of gynecological surgery (Y) | 185.61 | 0.014 |
| History of laparoscopy (Y) | 206.94 | 0.002 |
| History of previous ART (Y) | 191.08 | <0.001 |
| Previous pregnancy achieved |  | 0.004 |
| Naturally | 72.96 |  |
| After fertility treatment | 93.55 |  |
| Region** |  | 0.008 |
| Nabatiyeh | 212.5 |  |
| Beqaa | 170.97 |  |
| North | 220.75 |  |
| South | 174.46 |  |
| Mount Lebanon | 179.15 |  |
| Beirut | 147.03 |  |
| **Qualitative variables** | **Rs** | **p-value** |
| Duration of marriage | 0.156 | 0.004 |
| Number of cigarettes smoked/ day | -0.115 | 0.032 |
| Nargileh sessions per week | -0.115 | 0.032 |

* Mann-Whitney U test and Kruskal-Wallis H non-parametric tests

** Mann-Whitney U test: Beqaa vs North p=0.016, North vs South p=0.055, North vs Mount Lebanon p=0.023, and North vs Beirut p<0.001).

Variables that lacked significance with the practice score (living area p=0.235, health insurance p=0.804, occupation p=0.196, educational level p=0.342, monthly income p=0.383, cigarette smoking p=0.116, nargileh smoking p=0.341, alcohol consumption p=0.317, BMI p=0.586).
